# Life-threatening bleeding from gastric dieulafoy’s lesion in a pregnant woman with hellp syndrome: a case report and literature review

**DOI:** 10.1186/s12876-017-0646-1

**Published:** 2017-07-31

**Authors:** Chen Si, Zhu Xiuli, Xie Li, Jia Yong, Zhou Ying, Zhang Kaiguang

**Affiliations:** 1 Department of Gastroenterology, Affiliated Provincial Hospital of Anhui Medical University, Hefei, China; 2 Department of Obstetrics, Affiliated Provincial Hospital of Anhui Medical University, Hefei, China

**Keywords:** Dieulafoy’s lesion, Pregnancy, HELLP syndrome

## Abstract

**Background:**

Dieulafoy’s lesion (DL) is one of the rare causes of upper gastrointestional bleeding. This disease is characterized by small sub-mucosal arteriole that eroded the stomach mucosa and cause severe upper GI bleeding without obvious ulceration. The most common location is fundus area of stomach and usually affects patients over 50 years of age with multiple comorbidities.

**Case presentation:**

We report a case of life-threatening bleeding from DL during late pregnancy 31 weeks. Hemoclips were used twice through upper endoscopy with successful hemostasis. Unfortunately, she developed HELLP syndrome diagnosed 5 days after the GI bleeding was stopped. Her pregnancy had to be terminated with delivery of a premature infant. She recovered from her illness and discharged from hospital uneventfully. There is no current report in literature of DL in pregnant woman subsequently suffered HELLP syndrome.

**Conclusion:**

Endoscopic hemoclip application is an effective technique in the treatment of upper GI bleeding from DL. For this patient, laparoscopic surgery or combination therapy before pregnancy may have been a suitable treatment on preventing rebleeding.

## Background

Dieulafoy’s lesion (DL) is one of the rare, but potentially life-threatening causes of massive upper gastrointestinal (GI) bleeding. It accounts for about 1–2% of acute upper GI bleeding and occur more common in gastric fundic area at all age [1]. The diagnosis in pregnant women has not been reported. Due to the hemodynamic changes during pregnancy, DL is likely increase the morbidities and mortalities of pregnant women. Here we report a case that a pregnant woman who had massive upper GI hemorrhage from Dieulafoy’s lesion of stomach. She was treated successfully with hemoclips endoscopically with supportive care including fluid resuscitation, blood transfusion. Unfortunately, the patient eventually developed HELLP (hemolysis, elevated liver enzymes, and low platelets) syndrome. Termination of pregnancy prematurely to save the mother and baby’s life was the only option.

In 1982, Weinstein firstly described the HELLP syndrome which including hemolysis, elevated liver enzymes and low platelets [2]. The incidence of this syndrome is only 0.2%–0.6% in pregnant women and the exact etiology has not been clearly defined. There were case reports about Dieulafoy’s disease and HELLP syndrome occurred in women. To our knowledge, sequential occurrence of DL and HELLP syndrome were not reported so far.

## Case presentation

**Fig. 1:**
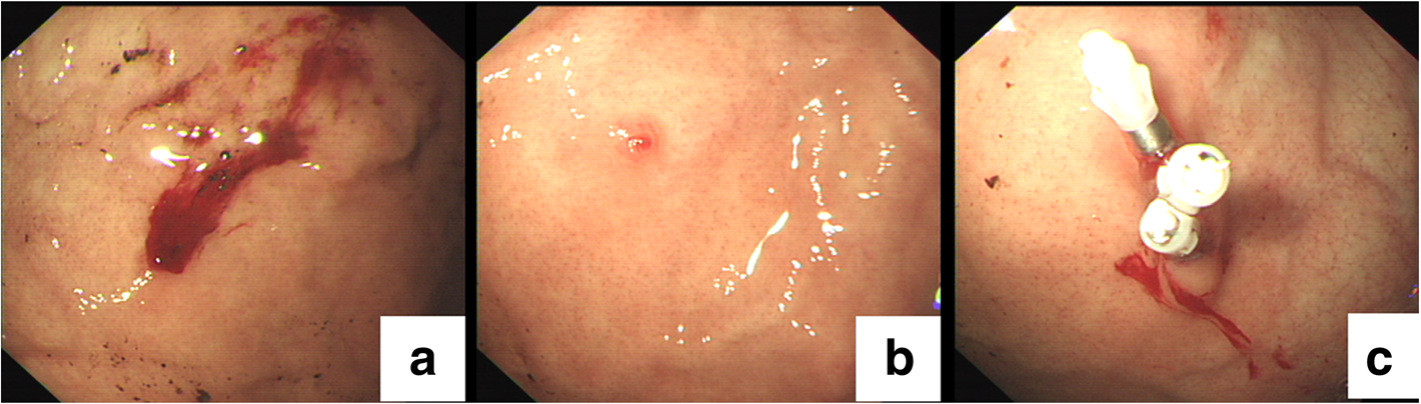
**a**, **b** GI endoscopy revealed active bleeding from gastric fundus and an exposed vessel protruded from a slight defect. **c** The lesion was treated with three hemoclips

A 27-year-old female patient was admitted to the gastroenterology department of our hospital due to hematemesis and melena. She was at 31 weeks and 4 days gestation. The patient had gastric DL diagnosed by esophagogastroduodenoscopy (EGD) 8 years ago when she presented with frequent hematemesis and black stools. EGD showed an blood vessel with bleeding over her gastric fundus about 6 cm from the gastroesophageal junction (Fig. 1). She had three episodes of upper GI bleeding from the same lesion without no overt symptoms.

**Fig. 2:**
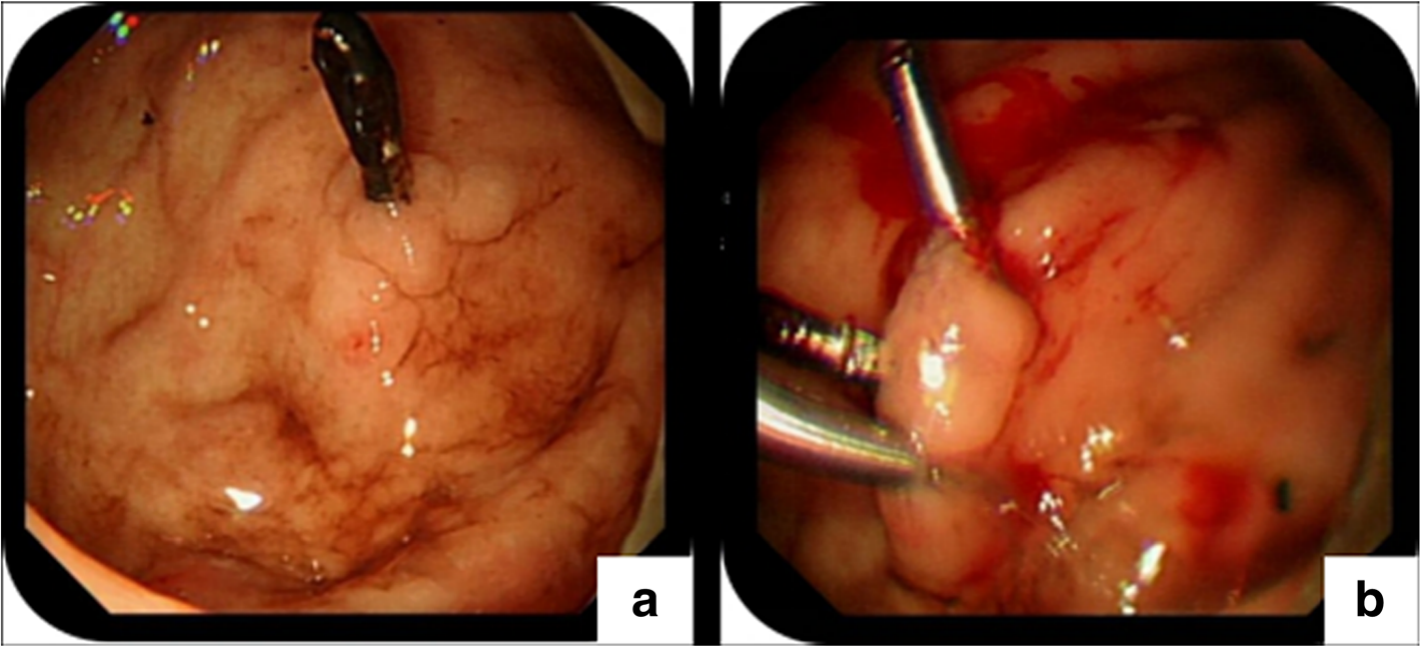
**a** The possible bleeding site adjacent to old lesions and hemoclips last used can be observed. **b** The lesion was treated with titanium clamhemoclips for prevention of recurrent bleeding

**Fig. 3:**
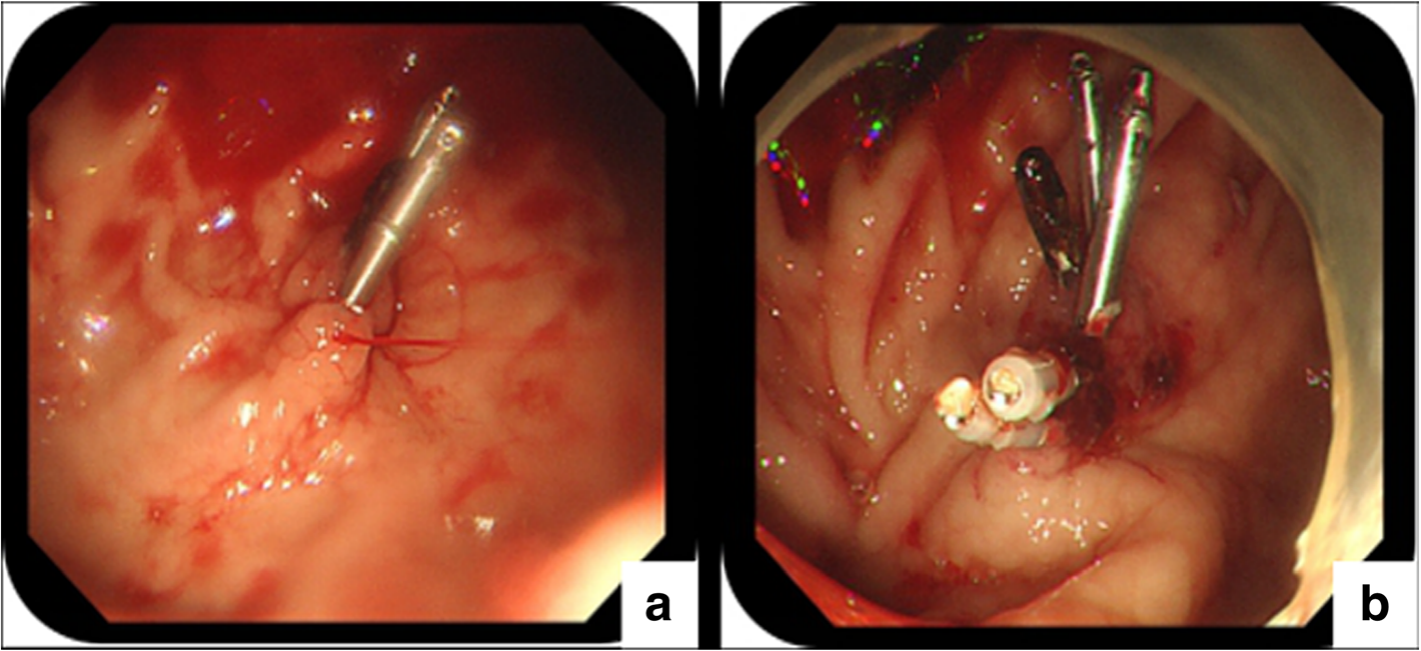
**a** A pulsatile bleeding in the upper stomach without underlying ulceration. **b** Resolution of hemorrhage following placement of hemoclips

In this case, emergency EGD (GIF Q260, Olympus Optical Co., Japan) revealed no active hemorrhage detected in upper digestive tract, other than the DL. The DL lesion in the fundus of stomach was treated with hemoclips for recurrent bleeding prophylaxis since she is having upper GI bleeding (Fig. 2). Unfortunately, massive hematemesis occurred again 2 days later and a second emergency gastroscopy was performed, which showed a spurting arterial bleeding without underlying ulceration close to the previous hemoclips. Hemoclips were applied again (Fig. 3). Finally, hemostasis based on endoscopic examination was achieved. However, even with blood transfusion support, the patient developed progressively anemia and thrombocytopenia without evidence of GI bleeding such as guaiac negative stools. Because of paroxysmal mild chest tightness and abdominal pains, Dexamethasone and magnesium sulfatate were administered intravenously to prevent eclampsia. Her laboratory examination showed: hemoglobin (HB), 62 g/L, platelet count, 19 × 10^9^/L, aspartate aminotransferase (AST) level and glutamic-pyruvic transaminase (ALT) were 136 IU/L and 252 IU/L respectively, serum total bilirubin, 55.7 μmol/L, lactate dehydrogenase (LDH), 1740 IU/L. Peripheral blood smear and elevated indirect bilirubin (38.5 μmol/L) revealed presence of schistocytes. Her blood pressure also increased to 155/91 mmHg. Meanwhile, there was signs of fetal distress and patient presented with acute abdominal pain. With evidence of acute hemolysis, thrombocytopenia and liver dysfunction, the pregnant woman was diagnosed with HELLP syndrome.

To prevent the further deterioration of mother and fatal condition, immediate delivery of the preterm baby by cesarean was recommended by hematology and gynecology consultation. She had uneventful surgery and discharged from hospital a week later. Her laboratory studies prior to discharge showed HB of 80 g/L and platelet count of 88 × 10^9^/L. Serum bilirubin, AST and ALT were all within normal limits. No recurrence of GI bleeding was noted within 1-month of outpatient follow-up. Premature baby was also discharged from hospital after delivery within 30 days showing no complications.

## Discussion and conclusion

DL is one of the rare causes of GI bleeding. It accounts for about 1.5% of all GI hemorrhages [3, 4]. Due to the subtlety of the lesion, endoscopic visual criteria have been established for diagnosis of DL [5]. In this case we observed active arterial spurting from a small (<3 mm) defect in the gastric mucosa. DL associated upper GI bleeding during late pregnancy is extraordinarily rare in clinical practice. There was only one case report of duodenal DL associated bleeding in the first trimester of pregnancy in literature (Table 1) [6]. It can occur anywhere in the gastrointestinal tract. Some lesions are extra-gastric including duodenum and esophageal [5, 6].

**Table 1:** Bleeding DL in pregnant woman: literature review

Author	GA at diagnosis (weeks)	Treatment	Complications	Follow-up (months)	Outcome
Benedetto et al.	9	Endoscopy with HCPs	no	1	Normal
Chen et al.	31	Endoscopy with HCPs	HELLP syndrome	2	Normal

*GA* gestational age, *HCPs* hemoclips

Although there is no consensus on the best techniques to treat DL, GI endoscopy is a vital tool [7, 8]. The common endoscopic treatment for DL associated GI bleeding include thermal probe, regional injection of epinephrine and mechanical methods [9–11]. The use of hemoclips proves to be easy and safe technique in treating DL GI bleeding and preventing rebleeding [11]. In our case, the pregnant woman suffered from pulsatile bleeding has been multiple hospitalizations for hemostasis which was controlled with emergency endoscopy two times. During late pregnancy, enlarged uterus can cause gastrointestinal compression leading to delayed gastric emptying. Retention of gastric juice and food may result in damage to the mucous membranes; thus, the risk of DL rebleeding may be higher in the third trimester.

Surgery is needed in about 3% to 16% of cases in patients with DL bleeding due to rebleeding after endoscopic therapies [12–14]. Laparoscopic surgery has been reported to treat patient with DL bleeding with advantages of minimally invasive intervention, but accurate localization of the bleeding site can be challenged [15]. For this patient rebleeding of DL occurred at the same site of stomach, so wide wedge resection of laparoscopic surgery with intraoperative endoscopy before pregnancy could have been a preferable treatment for patient with frequent recurrence of DL bleeding. Combined modality such as epinephrine injection and application of hemoclips therapy can achieve higher rate of initial hemostasis and lower rate of rebleeding when compared with monotherapy [12, 14]. It also may be a better option for our current case.

Even with the success of endoscopic therapy, massive hemorrhage has led to the progressive decline of PLT and eventually the HELLP syndrome occurred. It may be associated with massive hemorrhage and repeatedly erythrocyte transfusion. Moreover, hypoxia and heterologous related antigen during transfusion triggered immune reaction and inflammatory response that are both included in the etiology and pathogenesis of HELLP [16]. Damage to the vascular endothelial cell by activated platelets and other factors may cause thrombotic microangiopathy. This is considered to be the main pathogenetic mechanism of HELLP [16, 17].

The syndrome is typically observed in patients with severe preeclampsia. Although there was no severe preeclampsia in our case, laboratory results were consistent with the diagnosis of HELLP syndrome. The diagnostic criteria of the HELLP syndrome was initially proposed by Tennessee Classification System [18]. It includes presence of hemolysis, thrombocytopenia and hepatic changes with increased levels of enzymes. Pregnancy termination is still considered to be the best HELLP treatment method ensuring safety of the mother and infant [17, 18]. Current case presents nonspecific symptoms and stable delivery of the fetus by cesarean section. The mother and infant recovered uneventfully following effective treatment.

The patient with DL followed by HELLP syndrome is rare. Koji et al. have reported a case of HELLP syndrome with pituitary apoplexy [19]. Endoscopic therapy, especially hemoclips, is an initial and main treatment of bleeding caused by DL. However, both combined endoscopic therapies and surgery might be needed in some patients who are in a high risk of life-threatening rebleeding. Pathogenesis of HELLP syndrome in patient with DL is unclear. Massive GI hemorrhage and inflammatory response may be the triggering factors that lead to HELLP syndrome.

## Abbreviations

DL: Dieulafoy’s lesion
GI: Gastrointestinal
HELLP: H (hemolysis) EL (elevated liver enzymes) LP (low platelet count)
PLT: Platelet
EGD: Esophagogastroduodenoscopy
